# An Innovative Solution for Post-Consumer Footwear Waste: Nonwoven Fibrous Structures with Thermal and Acoustic Insulation Properties

**DOI:** 10.3390/ma18204765

**Published:** 2025-10-17

**Authors:** Diana I. Alves, Renato Guimarães, Sofia M. Costa, Nuno A. T. C. Fernandes, Óscar Carvalho, Raul Fangueiro, Diana P. Ferreira

**Affiliations:** 1 Textile Science and Technology Centre (2C2T), Department of Textile Engineering, University of Minho, Campus de Azurém, 4800-058 Guimarães, Portugal; renato.guimaraes@2c2t.uminho.pt (R.G.); sofiamcosta@2c2t.uminho.pt (S.M.C.); rfangueiro@det.uminho.pt (R.F.); 2 Center for Microelectromechanical Systems (CMEMS), Department of Mechanical Engineering, University of Minho, Campus de Azurém, 4800-058 Guimarães, Portugal; nnunofernandes@dem.uminho.pt (N.A.T.C.F.); oscar.carvalho@dem.uminho.pt (Ó.C.)

**Keywords:** acoustic insulation, circular economy, footwear mixture, leather, post-consumer waste, recycling, thermal insulation

## Abstract

**Highlights:**

**What are the main findings?**

- An eco-innovative material was developed from post-consumer footwear waste.
- The recycled nonwoven structures show good thermal and acoustic insulation properties.

**What is the implication of the main finding?**

- Footwear waste can be upcycled into sustainable insulation materials.
- Processing conditions allow tuning between mechanical strength and insulation per-formance.

**Abstract:**

With 23.4 billion pairs made and 22 billion discarded in 2023, post-consumer footwear waste is a major environmental challenge, demanding a shift toward circular economy practices. In this work, post-consumer footwear waste is repurposed into thermal/acoustic insulation materials for building construction, producing four needle-punched nonwovens (two of them compressed) composed of a post-consumer leather (30%) and footwear waste mixture (40%) with recycled polyester fibers. Nonwovens exhibited higher strain values (95.9 and 77.1% for leather residue and footwear mixture residue, respectively) but lower tensile strength (1694 and 104.9 kPa) and Young’s modulus (1767.8 and 136.10 kPa). The compressed nonwovens demonstrated higher tensile strength (7360 and 3559 kPa) and Young’s modulus values (12992 and 4020.4 kPa) and reduced strain (56.6 and 96.9%). The thermal conductivity results revealed that the nonwovens exhibited lower values (0.040 and 0.046 W/(m·K)), indicating better insulation performance when compared with their compressed counterparts (0.060 and 0.058 W/(m·K)). The nonwovens demonstrated high sound absorption at higher frequencies, reaching peak absorption coefficients of 0.917 and 0.995, ideal for acoustic insulation. The compressed nonwovens exhibited improved absorption at lower and mid-frequencies, with maximum values of 0.510 and 0.519. Given the current lack of applications for recycled materials derived from post-consumer footwear, the findings offer a novel approach to address their recycling.

## 1. Introduction

Post-consumer footwear waste is a major challenge for both the footwear industry and waste management [1]. In 2023, global footwear production reached a record-breaking 23.4 billion pairs [2,3,4]. This unprecedented high production volume is driven by the growing global population and the concept of fast-fashion, characterized by accelerated footwear/clothing design, manufacture, and distribution, allowing for rapid adaptation to shifting fashion trends and the release of new styles to the market within weeks [2,4,5]. As a result, an estimated 22 billion pairs of shoes were discarded in landfills in the same year [6].

Each stage of the life cycle of footwear has a significant environmental burden. Once discarded, footwear becomes post-consumer waste, the most pressing environmental burden in the footwear industry’s supply chain [1]. Raw material extraction requires substantial water, energy, and natural resources. Once extracted, materials undergo processing techniques such as spinning (for fibers), weaving (for textiles), and tanning (for leather). The assembly of the final product often requires adhesives, glues, stitching, and molding techniques. Even at the end of its useful life, a shoe remains harmful, as it undergoes landfilling or incineration processes. Cumulatively, these stages contribute to substantial environmental degradation, including waste accumulation; greenhouse gas emissions; resource depletion; and pollution of air, water, and soil. These impacts accelerate climate change and pose direct risks to ecosystems and human health [7,8,9].

Despite the severity of these impacts, post-consumer footwear waste remains largely excluded from existing recycling and circular economy strategies [4]. The complex composition of footwear, with up to 40 different materials [10,11], makes disassembly and material separation technically and economically challenging [1,12]. Footwear components can be divided into three categories: the upper, the lower, and the grindery. The upper part covers the front part of the foot and includes all footwear components above the sole. This part is usually stitched and attached to the insole and outsole of the shoe. The lower part of the shoe refers only to the sole and does not include the insole. The remaining footwear components that do not belong to either the upper or the lower part, such as eyelets, stiffener materials, and insoles, correspond to the grindery [4,13]. Footwear is mainly composed of leather (25%), polyurethane (17%), synthetic materials (38%), rubber (7%), and textiles (6%) [14]. Rubber can be either natural or synthetic, and while synthetic rubber offers greater chemical resistance and durability against temperature variations, it is not biodegradable like its natural counterpart. Both forms have environmental impacts during their production process, including deforestation, greenhouse gas emissions, and the release of volatile organic compounds, among others [15,16]. Synthetic materials, such as polyethylene and polyester, are also widely used in footwear [17]. Textiles are commonly used in the lining and upper parts of footwear and can include various materials such as cotton, nylon, viscose, polyester, and wool [18]. Both natural and synthetic textiles have environmental impacts, and natural fibers like cotton require large-scale pesticide use and are highly water-intensive, while synthetic fibers rely on non-renewable resources [4].

The footwear industry faces several challenges related to post-consumer footwear recycling, making this strategy inefficient [11,19]. Estimates suggest that less than 5% of post-consumer footwear is recycled at the end of its useful life [19]. Financial investment in post-consumer footwear recycling remains very limited, preventing this approach from being efficient and implemented on a large scale [20]. The extensive use of adhesives and stitching in shoe manufacturing makes the process of material separation even more complex. Most adhesives used to bond shoe components complicate the recycling process, making it difficult to separate and recover materials at the end of life [4,21], posing a significant challenge to achieve complete material separation and reclamation in an economically viable and sustainable manner [4]. Another challenge is the collection of post-consumer shoes, which is typically carried out through small-scale initiatives like shoe banks, charity shops, and recycling stations [14]. For a successful recovery chain, recycled shoe materials must compete with virgin materials in both performance and cost. Additionally, the volume of collected footwear waste must be substantial enough to justify and sustain the operational costs of a recycling facility. Furthermore, a key challenge in footwear recycling is developing a process capable of efficiently handling the diverse materials used in shoemaking [15]. Establishing an environmentally friendly system is complex and costly, often making it more expensive than conventional waste management methods like landfilling.

Despite the complexity of footwear waste, recycling remains the most attractive management strategy, as it enables the recovery of materials through collection, sorting, deconstruction, and reprocessing into new products, aligning with the principles of the circular economy [8,22,23]. In this context, the present study contributes to the development of new recycling strategies by repurposing post-consumer leather and diverse footwear waste into functional fibrous materials, expanding the possibilities for material recovery and reuse within the footwear industry.

Leather is one of the most widely used materials in footwear, primarily in the upper, sole, or lining. Its widespread use is attributed to its versatility; it can be stitched, molded, and waterproofed while maintaining durability and breathability [20]. However, leather also carries the highest environmental burden among footwear materials throughout its life cycle [1]. As reported by Joseph et al., the excessive use of chemicals in this industry leads to severe pollution in areas near and downstream from tanneries [24]. Based on data from over 776 million pairs of leather footwear, it is estimated that more than 2.25 million tons of chemicals are used in leather treatment, with a significant portion released into the environment [18]. Leather’s production involves numerous environmental burdens, from the cattle rearing phase to the slaughterhouse stage and, finally, the tannery stage, where hides undergo chemical treatments to achieve desirable properties [15]. Chromium-based mineral tannins are commonly used due to their faster processing time and ability to produce flexible, durable leather suitable for footwear applications. However, chromium is a heavy metal that, during the process, releases harmful pollutants into the air, including sulfides; volatile organic compounds; and particles, some of which are classified as carcinogenic [4].

Post-consumer leather waste is primarily valorized through pyrolysis, a thermal degradation process that breaks down organic materials in the absence of oxygen [1,11,21]. Kowalik-Klimczak et al. demonstrated the potential of converting leather residues into carbonized products suitable for pollutant adsorption in water and soil enrichment, and even as a graphite substitute [21]. Similarly, van Rensburg et al. highlighted the value of leather pyrolysis oils, which can be used in the formulation of fertilizers, adhesives, sealants, anti-adhesive agents, lubricants, and surface treatments [1]. Although pyrolysis offers a route for material recovery, it effectively terminates the lifecycle of leather or any other type of residue, limiting its potential within a circular economy framework, emphasizing the need for alternative approaches.

The typical recycling method involves shredding or granulating the footwear to break it down, followed by automated separation based on material properties. While these systems are generally effective at isolating materials with clearly distinct characteristics (like metals and certain plastics), they struggle with separating polymers and rubbers that have overlapping properties, as is common in footwear [19,25]. Alternatively, instead of focusing on separating individual components, it is possible to repurpose mixed footwear waste into new materials, as long as metal elements are removed beforehand [26]. This blended approach opens the door to automation, which is essential for scaling up recycling processes in a way that is both technologically practical and economically attractive. As such, it is possible to create new materials from mixed footwear waste, not only simplifying the recycling process but also enhancing its industrial feasibility.

A small fraction of footwear waste is currently reintroduced into the market using both destructive and non-destructive techniques. Destructive methods involve shredding the footwear into smaller fragments, which are then repurposed for low-value secondary applications such as playground surfacing, road base layers, or sound insulation. In contrast, non-destructive approaches aim to disassemble shoes in a controlled manner to recover individual components, preserving material quality and enabling their reuse in higher-value applications [14]. More specifically, in post-consumer footwear recycling, recovered components are used to produce new soles, laminates, composite materials, and epoxy coatings [21]. For example, Nike’s “Reuse-A-Shoe” program is one of the few post-consumer footwear recycling initiatives operating in the USA, UK, Japan, and Australia. Consumers drop off used shoes at collection points, which are then sent to recycling facilities, where they are shredded into “Nike Grind” material. This recycled material is used for surfacing tennis courts, running tracks, and playgrounds. The program has successfully diverted over 20 million pairs of athletic shoes from landfills [14]. Alexandrescu et al. developed and characterized biodegradable polymeric composites using natural rubber and protein waste derived from finished post-consumer leather. The experimental results were comparable to those obtained with composites made from virgin raw materials [27]. Despite these efforts, existing strategies remain limited in scope, scale, and material performance, underscoring the need for more efficient, scalable, and value-added approaches to repurpose post-consumer footwear waste.

Nonwoven fabrics represent a promising strategy for the valorization of waste materials. In the case of post-industrial footwear waste, only a few studies have demonstrated the feasibility of repurposing these residues into nonwoven structures for thermal and acoustic insulation panel applications [26]. In residential, commercial, and industrial construction alike, both acoustic and thermal insulation are critical for achieving occupant comfort and operational efficiency. Thermal insulation limits heat transfer through building envelopes, cutting heating and cooling loads and thus reducing energy consumption and carbon emissions (buildings account for 36% of global energy use and 39% of CO_2_) [28]. Acoustic insulation similarly improves indoor environmental quality by attenuating noise transmission, which is important in dense urban and industrial settings where excessive noise can cause stress, sleep disturbance, and other health issues [29]. However, many conventional insulation products (often fossil-derived foams or fibers) involve high embodied energy and poor end-of-life outcomes, which are difficult to recycle, persist for centuries in landfills or natural habitats, and can leach pollutants [30]. These environmental drawbacks have spurred research into greener insulation practices and materials. For example, recycled or bio-based insulators that meet thermal and acoustic performance requirements are increasingly sought to lower life cycle impacts [31]. Improving insulation technologies and practices is, therefore, necessary not only to meet energy-efficiency goals but also to reduce the ecological footprint of buildings, aligning construction with broader sustainability objectives.

Recent studies have emphasized the need to integrate waste valorization and sustainable material recovery strategies within construction and environmental engineering contexts. These studies underline how the reuse of discarded or low-value materials can significantly enhance resource efficiency, reduce embodied energy, and minimize environmental impacts throughout a material’s life cycle. By aligning recycling and circular economy principles with engineering design and manufacturing practices, such approaches not only mitigate waste accumulation but also contribute to resilient, low-carbon infrastructure systems [32]. However, current recycling strategies for footwear waste remain limited in scope and effectiveness, primarily due to the absence of scalable methods for reusing mixed footwear materials and the lack of comprehensive mechanical and physical characterization of recycled nonwoven structures derived from such waste. Addressing this research gap is crucial for advancing circular solutions that maintain functional performance while reducing environmental burden. Building upon this growing body of research, the present study introduces a novel strategy for repurposing post-consumer footwear waste into functional fibrous materials, demonstrating its potential as an innovative circular solution within the footwear and construction sectors.

In this work, post-consumer footwear waste was incorporated into nonwoven structures using the needle-punching method so it could be used for acoustic and thermal insulation. The materials used included leather and a mixture of footwear components. Subsequently, the nonwoven materials were subjected to high temperature and pressure through the compression molding technique to form compressed nonwoven structures. This approach is aligned with the principles of a circular economy, promoting a closed loop where materials are reused and recycled, contributing to a more sustainable and eco-friendly manufacturing process. By utilizing post-consumer footwear residues, this approach introduces innovation through a viable strategy that reduces landfill waste, minimizing the need for new raw materials, lowering energy consumption, and decreasing the environmental footprint of traditional manufacturing processes.

## 2. Materials and Methods

This study utilized two distinct types of post-consumer footwear waste: leather scraps and a heterogeneous footwear mixture. The leather materials were sourced from Lipor, the municipal waste management authority serving the Grande Porto area in Portugal. In contrast, the mixed footwear waste, already subjected to preliminary grinding, was supplied by the Centro Tecnológico do Calçado de Portugal.

### 2.1. Residue Characterization

To investigate the composition and thermal behavior of the waste materials, both were analyzed using Attenuated Total Reflectance Fourier Transform Infrared Spectroscopy (ATR-FTIR) and thermogravimetric analysis (TGA). ATR-FTIR spectra were collected using a SHIMADZU IRAffinity-1S spectrometer (Kyoto, Japan), operating in transmittance mode with a diamond ATR crystal. Each spectrum was recorded over the 4000–380 cm^−1^ range, averaging 45 scans at a resolution of 8 cm^−1^. Thermogravimetric measurements were performed on an STA 700 SCANSCI (Gaia, Portugal), with samples heated from 30 to 600 °C under a nitrogen atmosphere (200 mL/min flow rate) at a constant heating rate of 10 °C/min. To ensure comprehensive material identification, several ATR-FTIR and TGA tests were carried out on the mixture of footwear residue.

### 2.2. Residue Grinding

**Figure 1:**
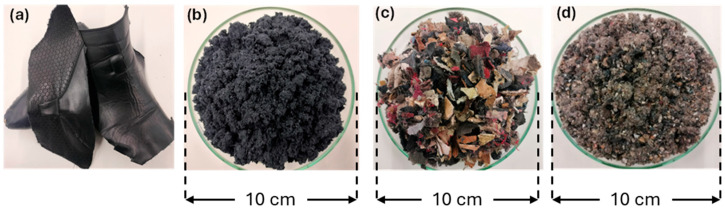
Post-consumer footwear waste before and after being ground using a Retsch SM300 grinder. (**a**) Leather before grinding; (**b**) leather after grinding; (**c**) footwear mixture before grinding; (**d**) footwear mixture after grinding.

The residues were mechanically processed using a Retsch SM300 (Düsseldorf, Germany) grinder (Figure 1). Leather scraps underwent four sequential grinding steps with decreasing mesh sizes: starting with a 10 × 10 mm sieve, followed by 4 × 4 mm, 2 × 2 mm, and finally 1 × 1 mm. Before processing the footwear mixture, metallic components were extracted using a neodymium magnet. This material was then ground in two stages: initially with a 10 × 10 mm sieve and subsequently with a 4 × 4 mm sieve.

### 2.3. Nonwoven Production

Nonwoven structures were produced using the needle-punching technique, employing a Cosmatex © Linea Campioni HL 500 (Benna, BI, Italy) textile machine integrated with an Automatex © (Terrebonne, Québec, Canada) needling system, forming a complete nonwoven production line. Since the footwear residues lacked a fibrous nature, incorporating a matrix material was necessary to enable web formation. Recycled polyester fibers, sourced from post-industrial textile waste, were selected as the matrix, reinforcing the study’s alignment with circular economy principles.

Initially, the recycled polyester fibers and footwear residues were manually blended before being fed into the carding stage, where they were arranged into a randomly oriented fibrous web. A cross-lapper was used to control the thickness of the web. The resulting web was then consolidated through needle-punching, involving repeated penetration by barbed needles. Each final sample consisted of a single layer of the polyester matrix incorporating the waste material. For leather-based samples, finer needles (15 × 18 × 38 × 3 mm) were employed, while for the more rigid footwear waste, thicker needles (15 × 16 × 32 × 3 mm) were necessary. The needling machine was operated at 300 strokes per minute.

To determine the optimal composition for nonwoven structures, different waste incorporation proportions (10%, 20%, 30%, and 40%) were tested. Each formulation was evaluated based on key criteria such as mass loss during processing, visual appearance, and structural integrity. The overarching goal of this study was to maximize the amount of waste integrated into the nonwoven material without compromising its quality.

In addition, the most promising nonwoven structures were further processed by compression molding to obtain densified and compacted samples, called compressed nonwoven structures, for comparison. Compression molding was carried out using a LabEcon 300 system (Fontijne Presses, Rotterdam, The Netherlands), with both samples subjected to uniform processing parameters: 220 °C temperature, an applied load of 60 kN, and a holding time of 4 min.

As such, four nonwoven structures were produced: samples A and B consist of 30% leather residue, with B subjected to compression molding; samples C and D consist of 40% footwear mixture residue, with D subjected to compression molding.

### 2.4. Scanning Electron Microscopy

All residues and nonwoven structures were analyzed using Scanning Electron Microscopy (SEM) to examine their surface morphology and cross-sections. The analyses were conducted with a FlexSEM 1000 II from Hitachi (Tokyo, Japan). Before the analysis, the samples were coated with an Au film for 60 s, using a Quorum MiniQS Sputter Coater (East Sussex, UK). SEM images were captured using secondary electron mode with an accelerated voltage of 5 kV.

### 2.5. Physical Properties and Air Permeability

The area weight was determined in accordance with ISO 9073-1:2023 [33]. For each nonwoven, three randomly selected samples were analyzed using an electronic balance, with results reported in g/m^2^. Thickness measurements were performed following ISO 9073-2 [34], based on ten random readings per sample.

Air permeability, defined as the rate at which air flows perpendicularly through the surface of a nonwoven material over time, was assessed according to ISO 9237:1995 [35]. Tests were conducted using a TEXTEST FX 3300 (Schwerzenbach, Switzerland) device, operating at a pressure differential of 200 Pa. Each sample underwent ten measurements using circular test areas of 20 cm^2^, with results expressed in l/m^2^/s.

### 2.6. Mechanical Properties

Mechanical characterization was performed through tensile testing using a Mecmesin MultiTest-I (West Sussex, United Kingdom) system with a 10 kN load cell. The nonwoven specimens were prepared and tested according to ISO 9073-3 [36], with dimensions of 5 cm width by 25 cm length, and a gauge length set at 20 cm. For the compressed materials, tensile tests adhered to ASTM D3039 standards [37], using smaller samples measuring 1 cm by 7.5 cm, and a 5 cm gauge length. During testing, all samples were oriented such that the applied force was parallel to the specimen’s longer axis. A total of five replicates per sample were tested, and the average values of tensile strength and strain were calculated from these repetitions.

### 2.7. Thermal Properties

Thermal behavior, including thermal conductivity (λ) and thermal resistance, was assessed using an Alambeta apparatus (Sensora, Liberec, Czech Republic). The test setup involves a heated metal plate maintained at 32 °C, which is brought into contact with the sample initially at room temperature (20 °C). Upon contact, a sudden temperature change occurs at the interface, and the device monitors the heat flow across the sample surface over time. Thermal conductivity represents the material’s capacity to conduct heat, defined as the heat transfer rate per unit area and temperature gradient, and is derived by dividing the sample’s thickness by its thermal conductivity [38]. Measurements followed the guidelines of ISO 5085-1 [39], with data collected from five distinct points on each specimen to ensure reliability. Thermal resistance values were determined using Equation (1).

(1) $$R=\frac{d}{k}$$

where R is the thermal resistance, d is the sample thickness, and k is the quantity of heat that passes in unit time through the area of the sample.

### 2.8. Acoustic Properties

Acoustic absorption was evaluated by measuring the sound absorption coefficient (α) following ISO 10534-2 [40]. Tests were performed using a custom-designed Kundt impedance tube with an internal diameter of 80 mm and a length of 410 mm. A probe was installed at 48 mm from the sound source, an 8 Ω speaker. The measurement system incorporated a Microflown probe (Enschede, The Netherlands), an MFPA-2 preamplifier (Microflown, Enschede, The Netherlands), a Scout 442 amplifier (Microflown, Enschede, The Netherlands), and a Microflown digital frequency analyzer (Enschede, The Netherlands). Circular samples of 120 mm diameter were examined over a frequency spectrum (300–2000 Hz). To investigate the effect of material thickness, certain specimens were stacked and tested in combination. Frequency data were divided into two intervals: low frequencies (≤500 Hz) and medium frequencies (500–2000 Hz). Each sample underwent five random measurements to ensure reliable acoustic property characterization. In order to compare the results with the commercially available solutions, a control sample was used that was made from an acoustic panel of polyurethane foam.

## 3. Results and Discussion

### 3.1. Residues Characterization

#### 3.1.1. Chemical and Thermal Characterization of Leather Residues

**Figure 2:**
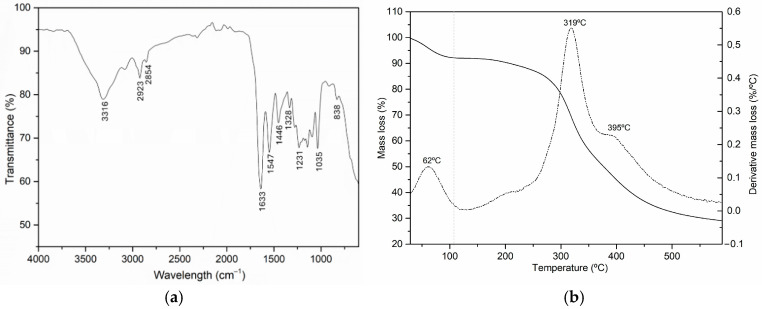
Characterization of leather residues showing the characteristic peaks in transmittance and derivative mass loss used to identify the material. (**a**) ATR-FTIR spectrum; (**b**) mass loss and derivative spectra.

The ATR-FTIR and TG/DTG spectra of the leather residue are shown in Figure 2. The ATR-FTIR spectrum is very similar to that of collagen, which is expected since it is the main component of leather. The intense band at 1633 cm^−1^ corresponds to C=O stretching, indicating the presence of amide I. The presence of amide II and amide III is confirmed by the bands at 1547 cm^−1^ (peptide bond) and 1231 cm^−1^ (collagen), respectively, which are associated with N-H bending and C-N stretching. The hydrogen bonds from the interaction between collagen and water are visible in the bands at 3316 cm^−1^ and 2923 cm^−1^, which are associated with OH/NH and NH stretching, respectively. Carbohydrate moieties in collagen proteoglycans are characterized by spectral bands at 1035 cm^−1^ and 838 cm^−1^, associated with C–O stretching and C–O–C stretching. The bands at 1446 cm^−1^ and 1328 cm^−1^ are attributable to CH_2_ and CH_3_ bending, referred to as aliphatic absorptions [41,42].

In Figure 2b, the thermal stability of leather residue is presented. Two main regions appeared in the temperature range from room temperature to 600 °C, corresponding to water removal and active pyrolysis in the main decomposition (active pyrolysis). The slight mass loss observed from room temperature to 108 °C is attributed to the evaporation of absorbed and bound water. The principal mass loss occurring between 250 °C and 600 °C is due to active pyrolysis, which is attributed to the decomposition of collagen. During the pyrolysis under a N_2_ atmosphere, various gases are released, including H_2_, CH_4_, CO, CO_2_, C_2_H_4_, C_2_H_6_, SO_2_, NH_3_, toluene, and other hydrocarbons [43]. In the differential mass loss spectrum as a function of temperature, a main peak is visible at 319 °C, accompanied by a shoulder at 395 °C. This shoulder indicates the presence of chromium in the composition of the leather [44].

#### 3.1.2. Chemical and Thermal Characterization of Footwear Mixture Residues

**Figure 3:**
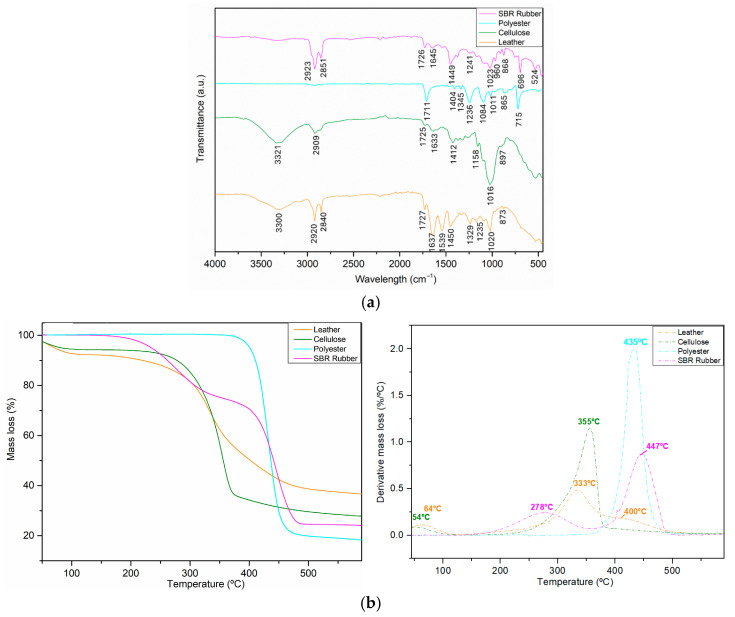
The chemical and thermal characterization of the footwear waste mixture reflecting the presence of several distinct residues. (**a**) ATR-FTIR and (**b**) TG/DTG spectra.

Different components of the footwear mixture were grouped according to their visual characteristics and individually characterized by ATR-FTIR (Figure 3a) and TGA (Figure 3b). The analysis revealed four distinct materials: leather, cellulose, polyester, and SBR rubber. The ATR-FTIR spectrum of leather is very similar to the previously presented spectrum (Figure 3a). The spectrum shown in green is characteristic of cellulose. The bands at 3321 cm^−1^ and 1633 cm^−1^ correspond to the OH bending of water. A minor band at 2909 cm^−1^ is associated with CH stretching. At 1412 cm^−1^, CH and OCH in-plane bending vibrations are visible. The intense band at 1016 cm^−1^ corresponds to C–O stretching. CO stretching, along with COC, C-CO, and C-CH deformation modes and stretching vibrations, is observed at 897 cm^−1^ [45,46]. The spectrum represented by the blue line closely resembles that of pure polyester. The intense band at 1711 cm^−1^ corresponds to carbonyl group stretching. The peaks at 1404 cm^−1^ and 1345 cm^−1^ are associated with C-H bending, corresponding to the aromatic ring and alkane. Also related to C-H bending, the band at 865 cm^−1^ and the intense band at 715 cm^−1^ are visible. The bands related to C-O stretching can be observed at 1236 cm^−1^, 1084 cm^−1^, and 1011 cm^−1^ [47,48]. The last spectrum, shown in pink, corresponds to SBR rubber. The bands at 2923 cm^−1^ and 2851 cm^−1^ correspond to C-H stretching from aliphatic CH_2_ and CH_3_ groups in the butadiene component. The C-H bending bands are visible at 1449 cm^−1^, associated with methyl groups, and at 868 cm^−1^ and 696 cm^−1^, related to the styrene rings. The bands at 1726 cm^−1^ and 1645 cm^−1^ correspond to carbonyl stretching and C=C stretching, respectively, from the styrene aromatic rings or the butadiene double bonds. The vinyl and trans-1,4-butadiene structures are confirmed by the bands at 1023 cm^−1^ and 960 cm^−1^, which are attributed to C-H out-of-plane deformation. The band at 524 cm^−1^ is likely related to C-C vibrations, possibly influenced by silica or carbon black, which are commonly added to SBR [49].

The thermogravimetric analysis (Figure 3b) corroborated the ATR-FTIR analysis. For leather residue, two regions were observed within the temperature range from room temperature to 600 °C. The principal mass loss, due to collagen decomposition, occurs between 240 °C and 550 °C. In the DTG spectrum, the three characteristic peaks of leather are visible at 64 °C, 333 °C, and 400 °C. This spectrum is very similar to the previous one obtained for leather (Figure 2b). Cellulose (green line) has two distinct mass loss regions: a minor loss up to 102 °C, attributed to water vaporization, and a more significant loss (60%) between 250 °C and 380 °C, corresponding to cellulose dehydration and decomposition, with a maximum degradation peak at 355 °C [50]. Polyester exhibits only one decomposition zone, with a significant mass loss of nearly 80%, occurring between 380 °C and 475 °C [51]. The major decomposition of polyester occurs at 435 °C. SBR rubber (rose line) also exhibits two mass loss regions: the first shows a 25% mass loss between 180 °C and 347 °C, while the second shows a 50% mass loss between 385 °C and 485 °C. This initial decomposition step at 278 °C is due to the degradation of butadiene or the breakdown of plasticizers and additives present in the rubber. The main degradation is associated with the thermal decomposition of the styrene component and the polymer backbone of SBR rubber, with the degradation peak at 447 °C [52].

#### 3.1.3. Morphological Characterization of the Residues

**Figure 4:**
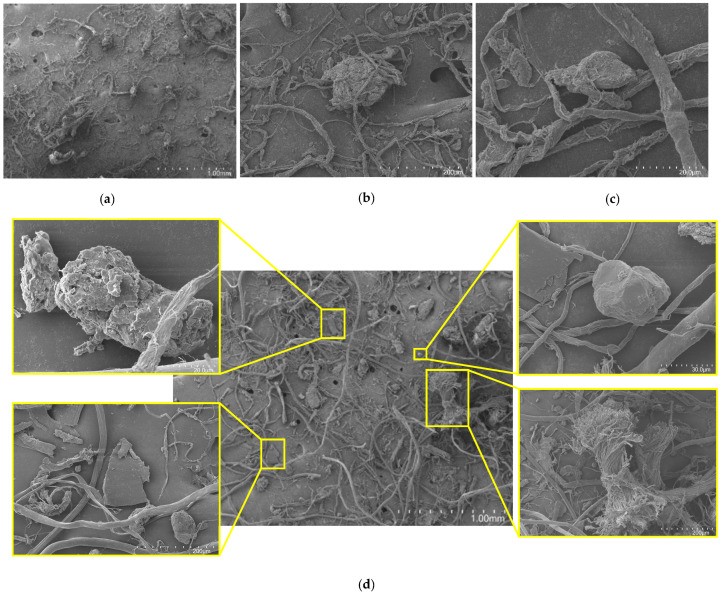
SEM images of the residues, depicting their morphological characteristics and dispersion. (**a**) Leather residue, 110×; (**b**) leather residue, 650×; (**c**) leather residue, 5000×; (**d**) footwear mixture.

The morphology of leather residues and the footwear mixture was evaluated by SEM analysis. In Figure 4a–c, a detailed analysis of the microstructure of leather residue is presented. The surface appears to be highly heterogeneous, with a complex network of fibrous structures and irregularly shaped particles. There is evidence of entangled fibers and small nodular formations in Figure 4a,b, suggesting the presence of collagen fibers and other organic matter typical of leather waste. Additionally, the fibrous network shows signs of degradation or mechanical wear. At the highest magnification, the intricate structure of individual fibers becomes more apparent, exhibiting rough surfaces and possible microfractures. This could be due to the nature of the residue itself, as it is post-consumer waste, or due to the material’s processing (grinding). These images confirm that leather residue can appear either as fiber or as an agglomerate.

The footwear mixture, as expected, presents highly distinct residues (Figure 4d). The central image reveals a highly irregular and heterogeneous surface with a complex network of fibrous structures and irregularly shaped particles, thus confirming the presence of various materials in this mixture. In the highlighted regions, a mixture of fibrous strands intertwined with fragmented particles can be observed, along with an agglomerated particle enmeshed with fibers (which could be residues from adhesives, fillers, or other additives used in footwear production) and non-fibrous materials. These images confirm the complexity of footwear materials, which influences their processing and the application of recycling processes.

### 3.2. Development and Characterization of Nonwoven Structures

**Figure 5:**
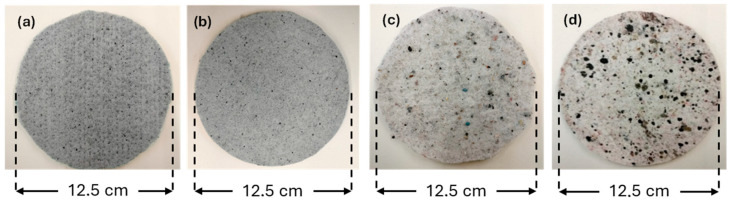
Nonwoven structures produced with post-consumer footwear waste. (**a**) Sample A (uncompressed leather nonwoven); (**b**) sample B (compressed leather nonwoven); (**c**) sample C (uncompressed footwear mixture nonwoven); (**d**) sample D (compressed footwear mixture nonwoven).

Nonwoven structures incorporating 30% leather waste and 40% post-consumer footwear mixture were produced (Table 1) and are illustrated in Figure 5, along with their corresponding compressed samples. These samples were subjected to a comprehensive characterization, including surface and cross-sectional morphology, physical properties and air permeability, mechanical, thermal, and acoustic performance.

#### 3.2.1. Surface and Cross-Section Morphological Characterization

**Figure 6:**
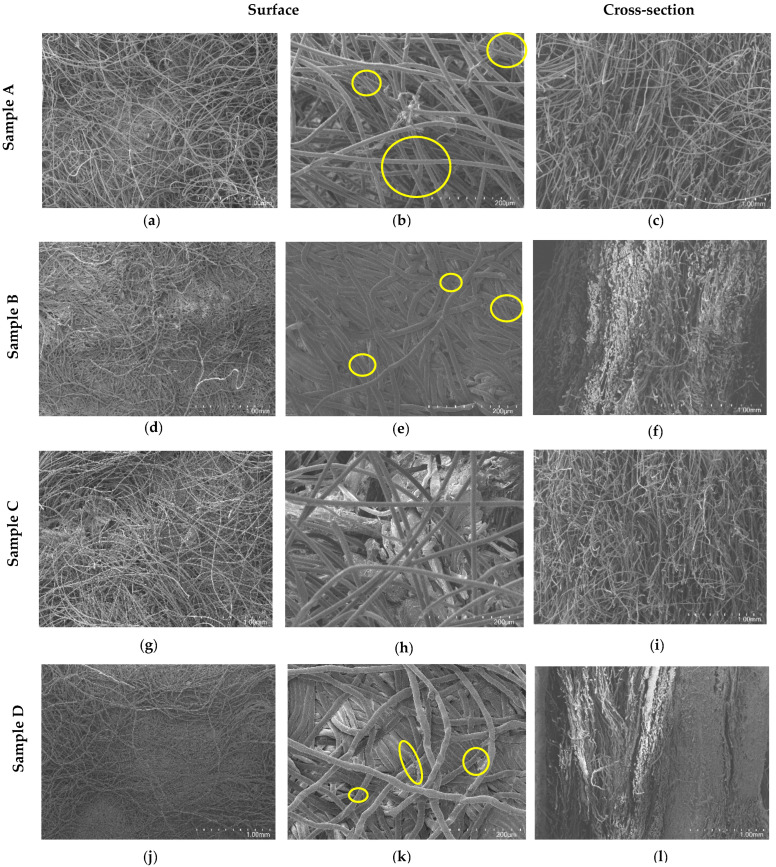
SEM images of surface and cross-sections of samples A, B, C, and D at different magnifications, illustrating their morphological features, fiber distribution, and structural compactness. (**a**) 110×; (**b**) 650×; (**c**) 110×; (**d**) 110×; (**e**) 650×; (**f**) 110×; (**g**) 110×; (**h**) 650×; (**i**) 110×; (**j**) 110×; (**k**) 650×; (**l**) 110×.

The sample surfaces were analyzed by SEM (Figure 6). The surface consists of non-aligned and disorganized fibers, with the fibers randomly arranged, as expected for nonwovens [7]. All nonwoven samples displayed randomly orientated fibers, and it is possible to observe good fiber bonding quality, as evidenced by the close contact between individual fibers, the absence of large gaps or loose ends, and the interlocking of fibers forming a coherent network. The SEM images reveal that fibers are entangled and connected at multiple points, which not only stabilizes the structure but also contributes to the mechanical integrity observed in tensile tests described in Section 3.2.3, where higher fiber interconnectivity corresponds to increased stiffness and load-bearing capacity. In the compressed structures, B and D, a significant compaction of the material is visible, with the fibers much closer together, reducing the empty spaces between the fibers, which leads to a decrease in porosity compared to the uncompressed nonwovens, A and C (Figure 6). In general, it is apparent that the residues (highlighted in yellow) help interconnect the fibers and fill the pores of the structure, making it more cohesive and resistant.

The cross-sectional images confirmed what had already been observed on their surfaces. In the case of the uncompressed samples, A and C, the fibers are completely disorganized, and there are many pores in the structure. In contrast, in the compressed samples, the fiber compaction results in a more organized structure with fewer empty spaces. Sample C has a much more heterogeneous composition than sample A, which is expected since sample C is composed of various waste materials, whereas sample A contains only leather waste.

#### 3.2.2. Physical Properties and Air Permeability

Table 2 shows the samples’ physical properties and air permeability. Sample A (leather residues) exhibited a lower area weight ratio and bulk density than the nonwoven compressed sample C (footwear mixture). This occurs because the leather nonwoven contains finer leather residues, resembling powder, whereas the footwear mixture includes larger residues. Area weight and bulk density increased in compressed nonwovens B and D compared to nonwovens A and C due to compression during manufacturing. The compressed samples had the same weight concentrated in a smaller volume, resulting in higher area weight and bulk density, as evidenced by the reduced thickness.

Understanding air permeability is crucial for optimizing materials used in thermal and acoustic insulation because it controls airflow and reduces energy loss. Lower air permeability (corresponding to higher airflow resistivity) enhances thermal insulation and sound absorption by increasing the viscous and thermal dissipation of thermal and acoustic energy within the porous structure, up to a certain point. Conversely, higher air permeability facilitates convective air flow, which can reduce thermal insulation performance, while lower air permeability helps retain heat and improve insulation [53]. For nonwoven structures (A and C), the air permeability values were much higher than those of compressed nonwoven samples (B and D), as expected from the SEM image analysis. The compression molding technique significantly reduces the samples’ porosity, thereby decreasing air permeability. Structures incorporating the footwear mixture exhibit slightly higher air permeability values than those containing leather. This outcome is expected since the larger particle size of the footwear residues creates more pores within the structure. The standard deviation values for air permeability were significantly higher in the nonwoven samples (A and C), reflecting the structural heterogeneity typical of the needle-punching process. In contrast, the compressed samples (B and D) showed low standard deviations, as compression helps to homogenize the fibrous structure. Among all samples, sample C exhibited the highest variation, likely due to the diverse composition of post-consumer footwear waste, which includes fibers and residues of varying sizes.

#### 3.2.3. Mechanical Properties

**Figure 7:**
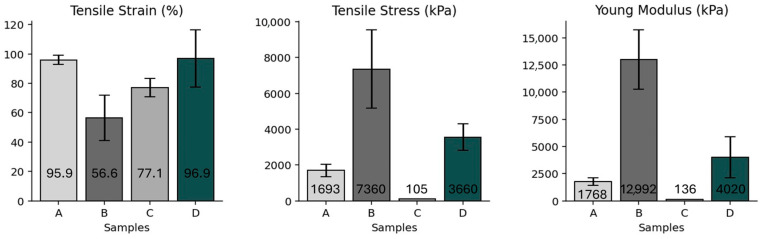
Mechanical characterization of the samples, presenting the tensile strength, the tensile strain, and Young’s modulus. The results highlight the influence of composition and processing on the mechanical performance of the footwear waste nonwovens.

The mechanical properties of the nonwoven panels play a critical role in determining their practical applicability, providing valuable insight into the material’s structural integrity and flexibility, ensuring a reliable performance in real-world applications. The respective strain (%), tensile strength (kPa), and Young’s modulus (kPa) values obtained for the samples are represented in Figure 7. Sample A exhibited a high strain of 95.9%, with moderate tensile strength (1694 kPa) and Young’s modulus (1767.8 kPa). These results indicate that the material is capable of large deformation but offers limited stiffness, likely due to the porous nonwoven structure and the intrinsic flexibility of the polyester fibers. In contrast, sample B, the compressed counterpart of sample A, showed a substantial increase in mechanical performance. Its tensile strength reached 7360 kPa, and Young’s modulus increased to 12,992 kPa, while strain decreased to 56.6%. The densification of the structure during compression enhances fiber–fiber contact and reduces voids, improving stiffness and load-bearing capacity, as evidenced in Figure 6, where SEM microscopy reveals a more compact fiber network, increased inter-fiber bonding, and fewer visible pores in the compressed samples compared to their uncompressed counterparts. The images highlight how fibers are closely intertwined, forming a cohesive structure that correlates with the observed increases in tensile strength and Young’s modulus. This confirms that compression significantly improves the mechanical integrity of leather-based nonwovens, making them more suitable for applications requiring enhanced strength and rigidity.

Sample C exhibited the lowest mechanical performance, with a tensile strength of only 104.9 kPa, a Young’s modulus of 136.1 kPa, and a strain of 77.1%. The weak performance is attributed to the uncompressed structure and the heterogeneity of the footwear waste, which contains materials with different sizes, textures, and chemical properties, resulting in reduced cohesion and fiber interlocking. When compressed, sample D showed notable improvements in both strength and stiffness. The tensile strength increased to 3559 kPa, and Young’s modulus rose to 4020.4 kPa, while maintaining a high strain of 96.9%. Although the improvements are less pronounced than in sample B, the enhanced performance still reflects the effectiveness of compression in reinforcing the fibrous structure. The variability in the mechanical response of sample D is likely influenced by the heterogeneous nature of the post-consumer footwear waste.

The standard deviations observed across the mechanical properties of the samples can be attributed to the intrinsic variability of the input materials, their innate anisotropy, and the processing techniques used. In nonwoven structures, samples A and C, the relatively low standard deviations in strain and modulus suggest a degree of consistency in fiber distribution and entanglement, despite the heterogeneity inherent to the needle-punching process. The inconsistent distribution of these materials within the nonwoven matrix leads to greater performance variability across test samples.

Compressed samples B and D displayed higher standard deviations, particularly in tensile strength and Young’s modulus. While compression increases overall structural integrity, it also amplifies the effect of localized material differences (such as rubber, polyester, or leather fragments) on the mechanical response. The densification process forces materials into closer contact, thus increasing fiber bonding quality by promoting greater inter-fiber entanglement, reducing void spaces, and enhancing the interlocking of individual fibers within the network. This improved connectivity stabilizes the structure and allows for more efficient load transfer between fibers, which contributes to the observed increases in tensile strength and stiffness. Sample B showed higher variability due to the natural heterogeneity of leather and its fine grinding, which can lead to localized accumulation within the structure (as shown in Figure 5). Thus, the standard deviations observed reflect the combined influence of material complexity and processing method, with greater variability arising in samples that undergo structural compaction.

Overall, the results demonstrate that compression significantly enhances the mechanical properties of nonwoven structures, particularly in samples with higher leather content. While diverse footwear waste also benefits from compression, its complex composition leads to more variable outcomes. These findings underscore the importance of both material composition and processing method in tailoring mechanical behavior for potential applications in footwear components or other structural uses.

The concept of reinforcing structural materials with waste-derived fibers has also been explored in the construction industry. Recent studies have demonstrated that incorporating macro-synthetic fibers or recycled e-waste aggregates into concrete can improve mechanical properties, such as tensile, flexural, and compressive strength, while also promoting sustainability [54,55]. These findings suggest that nonwoven panels derived from post-consumer footwear waste (either in its uncompressed or compressed form) could potentially serve as alternative fiber reinforcement in composite materials, including concrete or polymer composites. The high tensile strength, strain capacity, and structural integrity observed in samples B and D indicate that these waste-derived nonwovens may not only contribute to mechanical reinforcement but also support eco-friendly design strategies by repurposing post-consumer waste streams, offering a novel pathway for integrating circular economy principles into construction materials.

These nonwoven samples, both in uncompressed and compressed configurations, provide mechanically robust solutions while repurposing post-consumer footwear waste, offering a sustainable alternative to conventional structural or cushioning materials that often rely on highly polluting polymers or composites, such as polyurethane foams, polyethylene, polypropylene, and rubber-based synthetic materials [56]. This combination of mechanical performance and eco-friendly material sourcing demonstrates that waste-derived nonwovens can meet real-world structural demands while reducing environmental impact.

#### 3.2.4. Thermal Properties

Thermal resistance and thermal conductivity are fundamental parameters for evaluating a material’s thermal insulation performance, with lower thermal conductivity resulting in higher thermal insulation, and, conversely, higher thermal resistance, indicating higher thermal insulation. The thermal conductivity values (Table 3) are within the range of values for other nonwoven structures and commercial insulation materials (0.020–0.068 W/(m·K)) [57]. Compression molding increases thermal conductivity and decreases thermal resistance in both nonwoven samples. For the leather nonwoven (sample A), thermal conductivity rises from 0.040 to 0.060 W/(m·K), and thermal resistance drops from 0.110 to 0.069 m^2^·K/W. For the footwear mixture nonwoven (sample C), thermal conductivity increases from 0.046 to 0.058 W/(m·K), and thermal resistance decreases from 0.127 to 0.061 m^2^·K/W. This behavior is expected because compression reduces the material’s porosity, enhancing heat transfer pathways and lowering the resistance to heat flow.

Taking into account our previous study [26], it is possible to see that the obtained thermal properties are comparable with the previous samples, staying in the same range (0.37–0.41 for nonwovens and 0.60–0.70 for compresses samples). When comparing with other authors, uncompressed samples A and C are within the lower to mid-range of reported values, indicating effective insulation performance, which typically falls in the order of 0.03–0.057 [58,59,60]. Compression increases thermal conductivity slightly (0.058–0.060 W/(m·K)), consistent with the literature trends where densification reduces porosity and enhances heat transfer pathways (0.026–0.17 [61,62,63,64]). Overall, the thermal performance of both uncompressed- and compressed-footwear-derived nonwovens aligns with conventional nonwoven insulation materials, confirming their applicability for thermal insulation purposes.

Compared to rock wool, a common commercial thermal insulator with a thermal conductivity of 0.033–0.045 W/(m·K) [26], sample A falls within the same range, demonstrating competitive thermal performance. Sample C slightly exceeds this range, while samples B and D, due to their denser structure, exhibit higher thermal conductivity and are less suitable for thermal insulation, although they all fall within the aforementioned commercial values.

Sample A has lower thermal conductivity (0.040 W/(m·K)) compared with the footwear mixture nonwoven, sample C (0.046 W/(m·K)), likely due to larger residues creating more direct heat transfer paths. However, its thermal resistance is also higher because the larger pores trap more air, which is a poor conductor of heat. After compression molding, the difference in thermal conductivity between the two materials becomes smaller (0.060 vs. 0.058 W/(m·K)), suggesting that compressing the samples reduces the effect of particle size on heat transfer. However, the compressed footwear nonwoven still shows lower thermal resistance (0.061 m^2^·K/W) compared to compressed leather (0.069 m^2^·K/W), indicating that larger particles create fewer insulating air pockets even after compression. These results are consistent with the air permeability values, where structures with higher permeability exhibit lower thermal conductivity. The nonwoven samples (A and C) exhibited generally low standard deviations in thermal resistance, despite their heterogeneous structure. This suggests that porosity, which plays a dominant role in thermal insulation for uncompressed materials [65], remained relatively consistent across replicates. In contrast, the compressed samples (B and D) showed a shift in the insulation mechanism. Compression significantly reduces porosity, making the thermal resistance more dependent on the intrinsic thermal properties of the materials rather than air-filled voids. As a result, sample D displayed a high standard deviation, likely due to its composition of diverse post-consumer footwear waste. The variability in material types and properties (depending on the specific mixture present in each sample) led to greater inconsistency in thermal performance.

These findings highlight that the waste-derived samples offer competitive thermal insulation properties, comparable to commercial materials such as rock wool. By repurposing leather and footwear mixture residues from post-consumer footwear, these materials provide an environmentally friendly alternative to conventional thermal insulators that rely on highly polluting compounds, such as polyurethane foams, polystyrene foams, fiberglass, mineral wool, and phenolic/melamine foams [26], supporting sustainable building and industrial applications.

#### 3.2.5. Acoustic Properties

**Figure 8:**
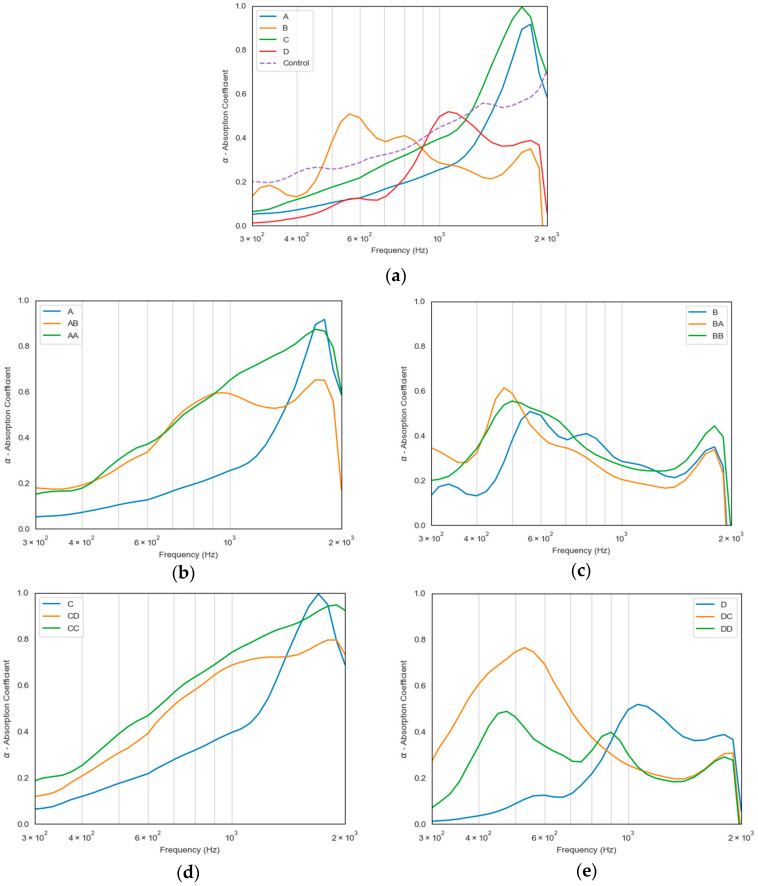
Frequency-dependent sound absorption coefficient of the samples, highlighting differences in acoustic performance related to material composition and structure in (**a**) single samples A–D; (**b**) coupling of sample A; (**c**) coupling of sample B; (**d**) coupling of sample C; (**e**) coupling of sample D.

The acoustic properties of the single samples were analyzed across a frequency range of 300–2000 Hz and are specified in Figure 8a. Sample A (nonwoven—30% leather) exhibits a steady increase in absorption with frequency, peaking at 1900 Hz, with a maximum of α = 0.917, being inefficient at lower frequencies but highly efficient at higher frequencies. When compressed (sample B), the material demonstrates improved absorption at lower frequencies (with a peak of α = 0.510 at 550 Hz) but a fluctuating lower trend at higher frequencies. Similarly, sample C follows a comparable pattern to A but with slightly lower absorption values (a maximum of α = 0.995 at 1800 Hz). However, its compressed counterpart, sample D, shows significantly higher absorption at mid-range frequencies (with a maximum α = 0.519 at 1100 Hz) but lower performance at higher frequencies compared to sample C. Compared to the control sample, all experimental samples, especially A and C, demonstrated superior sound absorption at high frequencies, with α values exceeding 0.9, while the control plateaued below 0.7. At mid-frequencies, samples B and D outperformed the control as well, highlighting the positive effect of compression on absorption at around 500–1100 Hz. These findings suggest that compression enhances sound absorption at lower frequencies, while uncompressed materials may perform better at higher frequencies. This trend was observed for samples A and B, in agreement with the results of our previous study [26]. However, samples C and D do not follow the same pattern, with the compressed sample (D) exhibiting reduced performance at both low and high frequencies, while showing a pronounced peak around 1000 Hz. Therefore, samples A and C are ideal for high-frequency and broadband acoustic insulation, suitable for architectural and consumer applications. Samples B and D, due to their mid-frequency performance and structural density, are better suited for technical or sound-absorption uses, especially where some mechanical strength is also beneficial.

In lower-frequency ranges, sound waves have longer wavelengths, and the denser structure results in better impedance matching, allowing more energy to be absorbed rather than reflected. As such, compression likely boosts sound absorption at lower frequencies due to increased material density and reduced pore size, which enhances energy dissipation through friction and viscous losses. However, higher-frequency sound waves, which have shorter wavelengths, rely more on internal pores and fiber interactions to dissipate energy. At higher frequencies, compression can reduce the effectiveness of absorption because the material’s porosity and internal air pockets decrease. When the structure is too compact, the sound waves may reflect more instead of penetrating and getting absorbed, leading to lower absorption coefficients.

Compared to previous research [26] using footwear polyester waste combined with recycled polyester, a similar trend was observed in the uncompressed samples, where higher absorption coefficients were recorded at higher frequencies. However, this study indicates that samples A and C demonstrate superior performance compared to the earlier findings. Notably, at lower frequencies, sample B exhibited good absorption, a behavior not reported in the previous study. This suggests that absorption at lower frequencies is primarily influenced by material composition and density, whereas at higher frequencies, absorption is also affected by material properties but is predominantly governed by the porosity of the material.

To further enhance acoustic performance, a coupling strategy was explored by layering different sample combinations. This approach aims to merge the complementary properties of nonwoven and compressed structures to achieve broader and more efficient sound absorption. The sound absorption coefficient observed by the coupling of the samples is demonstrated in Figure 8b–e. For sample A, the AA pairing is highly effective, delivering improved absorption across nearly the entire frequency range, particularly in the mid-to-high frequencies. The AB combination also performs well but does not match the efficiency of AA, likely due to the compressed B layer limiting its overall absorption capacity at higher frequencies. Lastly, sample B exhibits varying results, with BA performing well in mid-frequencies but showing slight weaknesses at higher frequencies. Meanwhile, BB improves absorption at high frequencies but does not perform as effectively as other combinations overall. The coupling of different samples reveals distinct absorption trends across the frequency spectrum. For sample C, the CC combination shows a significant improvement in absorption at all frequencies, particularly in the mid-to-high range, making it the most effective pairing. The CD combination also enhances absorption, though to a lesser extent, suggesting that the compressed D layer might limit performance at higher frequencies. In the case of sample D, the DC combination provides a noticeable boost in absorption, especially at lower and mid-frequencies, whereas DD exhibits inconsistent behavior with dips in absorption, indicating that excessive compression may reduce effectiveness.

The best coupling choices are CC and AA, as they provide the most consistent and significant absorption improvements. DC and AB also show potential, particularly for enhancing mid-frequencies and low frequencies. However, DD and BB are less effective, suggesting that excessive compression may hinder performance rather than enhance it. At mid-frequency (~500 Hz), the literature values range from 0.220 to 0.680. In this range, samples B (~0.45) and C (~0.25) fall within the expected values, whereas samples A (~0.12) and D (~0.15) underperform, and the control (~0.22) sits at the lower boundary. At high frequencies (1000–2000 Hz), the literature reports values from 0 to 1; all samples and the control fall within this range, with sample C (~1.0) reaching the upper limit. Overall, mid-frequency performance is strongest for B and C, while high-frequency absorption aligns well with the literature expectations for all samples [58,59,60,61,62,63,64]. Both single and coupled configurations demonstrate suitable acoustic insulation properties, repurposing waste materials such as leather and footwear mixture from discarded footwear, instead of relying on conventional commercial solutions that use highly polluting compounds like polyurethane foams, fiberglass, and mineral wool [26], while couplings provide opportunities for optimizing absorption across specific frequency ranges for tailored architectural, consumer, and technical applications.

## 4. Conclusions

Post-consumer waste was effectively integrated into recycled structures, demonstrating high efficiency as common thermal and acoustic insulation materials. The developed nonwoven and compressed nonwoven structures were produced using recycled polyester, incorporating either 30% leather waste or 40% footwear waste, both derived from post-consumer waste. These structures exhibited promising performance, with Young’s modulus ranging from 161 to 10,891 kPa and thermal conductivity values between 0.0340 and 0.0600 W/(m·K). Uncompressed samples achieved high sound absorption coefficients (α = 0.910–0.995) at higher frequencies, while sample B demonstrated effective absorption (α = 0.510–0.519) in the medium-to-low-frequency range. Additionally, coupling these structures further extended their absorption capabilities, highlighting their potential adaptability. To further characterize these materials, additional studies should focus on vibrational behavior, viscoelastic properties, impact resistance, flammability, and geometric influences, and additional tests should be carried out according to final use and overall commercial feasibility through life cycle assessment. The incorporation of recycled materials into construction applications reinforces the principles of a circular economy, reducing waste and promoting sustainability for future generations.

## Figures and Tables

**Table 1:** Sample composition.

Sample	Residue	% Residue	Compression Molding
A	Leather	30%	No
B	Leather		Yes
C	Footwear mixture	40%	No
D	Footwear mixture		Yes

**Table 2:** Physical properties and air permeability of samples.

Sample	Thickness (m)	Area Weight (kg/m^2^)	Bulk Density (kg/m^3^)	Air Permeability (l × (m^2^/s))
A	(4.40 ± 0.19)·10^−3^	0.160	36.3 ± 1.5	687 ± 34
B	(4.18 ± 0.04)·10^−3^	2.40	574.0 ± 6.0	9.24 ± 1.5
C	(5.94 ± 0.25)·10^−3^	0.490	82.4 ± 3.2	741 ± 151
D	(5.40 ± 0.05)·10^−3^	2.40	444.0 ± 4.0	14.47 ± 1.51

**Table 3:** Thermal properties of the samples.

Sample	Thermal Conductivity (W/(m·K))	Thermal Resistance (m^2^·K/W)
A	0.040 ÷ 0.007	0.110 ÷ 0.004
B	0.060 ÷ 0.002	0.069 ÷ 0.002
C	0.046 ÷ 0.003	0.127 ÷ 0.002
D	0.058 ÷ 0.003	0.061 ÷ 0.005
Rock Wool	0.039 ÷ 0.006	---

## Data Availability

The original contributions presented in this study are included in the article. Further inquiries can be directed to the corresponding authors.
